# Long-Term Ambient Air Pollution Exposure and the Risk of Cardiovascular and Cerebrovascular Diseases in Rural Chinese Populations: 10-Year Follow-Up of a Multicenter Prospective Cohort Study

**DOI:** 10.2196/81218

**Published:** 2025-11-28

**Authors:** Yaqi Zhao, Xuefang Cao, Jiang Du, Aiwei He, Jun Liang, Weitao Duan, Yuanzhi Di, Yijun He, Boxuan Feng, Linyu Shen, Juanjuan Huang, Zihan Li, Jianguo Liang, Hongzhi Li, Zisen Liu, Fang Liu, Shumin Yang, Zuhui Xu, Bin Zhang, Jiaoxia Yan, Yanchun Liang, Rong Liu, Fei Shen, Qi Jin, Henan Xin, Lei Gao

**Affiliations:** 1 NHC Key Laboratory of Systems Biology of Pathogens, National Institute of Pathogen Biology, and Center for Tuberculosis Research, Chinese Academy of Medical Sciences & Peking Union Medical College, No.16 Tianrong Street, Beijing, 102629, China, 86 13311185615; 2 Key Laboratory of Pathogen Infection Prevention and Control (Ministry of Education), National Institute of Pathogen Biology, Chinese Academy of Medical Sciences & Peking Union Medical College, Beijing, China; 3 Tuberculosis Prevention and Control Department, Gansu Provincial Center for Disease Control and Prevention, Lanzhou, China; 4 Tuberculosis Prevention and Control Department, Hunan Provincial Institute of Tuberculosis Prevention and Control, Changsha, Human, China; 5 Tuberculosis Prevention and Control Department, Zhongmu County Center for Disease Control and Prevention, Zhongmu, China; 6 Zhengzhou Sixth People’s Hospital, Zhengzhou, Henan, China; 7 Tuberculosis Prevention and Control Department, Longxi County Center for Disease Control and Prevention, Longxi, China; 8 Tuberculosis Prevention and Control Department, Xiangtan County Center for Disease Control and Prevention, Xiangtan, China

**Keywords:** cardiovascular and cerebrovascular diseases, particulate matter, air pollution, prospective study, Cox proportional hazards model

## Abstract

**Background:**

Long-term follow-up studies investigating the relationship between ambient air pollution and cardiovascular and cerebrovascular diseases (CVD) in rural Chinese populations remain limited.

**Objective:**

This study aimed to investigate the impact of prolonged exposure to particulate matter with aerodynamic diameter ≤2.5 μm (PM_2.5_) on CVD in rural areas of China.

**Methods:**

On the basis of a multicenter population-based prospective study, adult rural residents (aged ≥15 y) from 3 study sites (ie, Xiangtan, Hunan Province; Longxi, Gansu Province; and Zhongmu, Henan Province) with different PM_2.5_ exposure levels were tracked for the incidence of CVD events between 2013 and 2023. The relationship was assessed by applying the Cox proportional hazards model and a trend test.

**Results:**

A total of 15,502 participants were included in the study. During the 10-year follow-up period, for every 1 μg/m^3^ increase in PM_2.5_, the risks of CVD, ischemic stroke, coronary heart disease, acute coronary syndrome, and intracerebral hemorrhage increased by 5% (hazard ratio [HR] 1.05, 95% CI 1.04‐1.06), 7% (HR 1.07, 95% CI 1.06‐1.08), 8% (HR 1.08, 95% CI 1.07‐1.09), 9% (HR 1.09, 95% CI 1.06‐1.11), and 10% (HR 1.10, 95% CI 1.07‐1.14), respectively. Furthermore, the risk in the high exposure group (Q4) was found to be significantly higher than that in the low exposure group (Q1; *P* for trend <.001). The subgroup analysis indicated that the risk of CVD was higher among older people compared to individuals aged <60 years, and the interaction effect was statistically significant (interaction *P* value=.03).

**Conclusions:**

Our results indicate that long-term exposure to PM_2.5_ significantly increases the risk of CVD in rural areas of China and shows regional differences. This finding may deepen our understanding of the potential public health risks associated with PM_2.5_ exposure and emphasize the crucial role of environmental governance in promoting public health outcomes.

## Introduction

### Background

Ambient air pollution, considered the most significant environmental risk factor for global mortality and morbidity, particularly particulate matter with aerodynamic diameter ≤2.5 μm (PM_2.5_) with an aerodynamic diameter of less than 2.5 micrometers, has emerged as a significant global public health concern [1]. The Global Burden of Disease report indicates that in 2019, air pollution was responsible for the deaths of 9 million people worldwide, with 61.9% of these fatalities due to increased mortality from cardiovascular and cerebrovascular diseases (CVD) [2]. Furthermore, there are significant variations in air pollution standards worldwide [3]. The limits in China and India are considerably higher than those in high-income nations (at 35 and 40 μg/m^3^, respectively). Even in countries with lower levels of air pollution, there is evidence indicating a correlation between air pollution and cardiovascular health [4]. Early large-scale cohort studies reported the impact of long-term exposure to air pollution on CVD events in Europe and the United States. The environmental PM_2.5_ exposure levels were generally <35 µg/m^3^ [5,6]. A study of the European Study of Cohorts for Air Pollution Effects project [7] revealed that for every 5 μg/m^3^ increase in PM_2.5_ concentration, there was a 13% rise in the risk of coronary events. A cohort study conducted in the United States with 1,934,453 older participants found that for every 1 μg/m^3^ increase in PM_2.5_, the risk of transient cerebral ischemia and heart failure episodes increased by 3.5% and 1.9%, respectively [8]. This phenomenon may be attributed to the capacity of smaller particles to penetrate deeper lung regions and enter the bloodstream, thereby inducing systemic inflammation, oxidative stress, and endothelial dysfunction [9]. Consequently, this exposure increases the susceptibility of the CVD systems to the detrimental effects of these particles [10]. However, in many countries, particularly in low- and middle-income countries, the long-term effects of air pollution on health have not been thoroughly investigated [3]. In China, particularly in rural areas, there are numerous instances of biomass burning and coal-fired power plants [4]. The PM_2.5_ exposure levels in these rural areas are significantly higher than those in urban areas, far exceeding the World Health Organization’s Air Quality Guidelines [3]. However, epidemiological studies that link long-term air pollution in rural China to specific CVD are limited in scope.

### Objectives

This research aims to conduct a multicenter, population-based prospective study to explore the long-term association between PM_2.5_ exposure and the risks of CVD among rural residents from different regions. The findings of this study may contribute to a deeper understanding of the impact of air pollution on health and provide a more robust scientific basis for the development of public health policies in China.

## Methods

### Study Design and Participants

A population-based multicenter cohort study was initiated in 2013 and was a 10-year follow-up survey of registered residents at 3 study sites using a closed cohort design [11] (ie, Xiangtan, Hunan Province; Longxi, Gansu Province; and Zhongmu, Henan Province) between October 1, 2023, and January 31, 2024, to track the occurrence of CVD. This cohort study was jointly organized by the Institute of Virology at the Chinese Academy of Medical Sciences and the Chinese Center for Disease Control and Prevention. It was divided into 2 phases and spanned a duration of 10 years, from 2013 to 2023. During the initial phase, we gathered the participants’ sociodemographic information and disease history. In the second phase, which took place in 2014, 2015, 2018, and 2023, we monitored their disease onset status and corroborated and refined the data through the local chronic disease management system (CDMS). The inclusion criteria for the research participants were as follows: birth date before June 1, 1998 (age ≥15 y); possession of a household registration or residence permit for the village; continuous residence at the research sites for 6 months or longer in the past year; the ability to complete the investigations during the research period; and the provision of voluntary written informed consent. The exclusion criteria were individuals without a residence address at the time of the baseline survey, those lost to follow-up, and those who became pregnant. All eligible current residents living in the 3 selected research sites were included in the 10-year follow-up survey.

### Ethical Considerations

The study protocol was approved by the ethics committees of the Institute of Pathogen Biology, Chinese Academy of Medical Sciences, Beijing, China (approval number IPB-2023‐35). Written informed consent was obtained from all study participants, and all participants have the right to withdraw at any time. The study provides breakfast and transportation subsidies to the participants.

### Procedure

In this study, measures were implemented to ensure the quality and comparability of data across the 3 research sites. These included standardizing the research protocol, providing uniform training for researchers, and applying consistent disease diagnostic criteria.

Sociodemographic data for each research participant were systematically collected using standardized questionnaires administered by trained interviewers. The data included age, gender, educational attainment, smoking status, alcohol consumption status, weight, height, marital status, per capita household income for 2013 (calculated by dividing the total household income by the number of family members) [12], and a history of hypertension. Household income per capita was categorized based on the national mean level in 2010 (6000 RMB [US $887]) [13]. BMI was categorized as underweight (<18.5 kg/m^2^), normal weight (≥18.5 kg/m^2^ to <24.0 kg/m^2^), and overweight (≥24.0 kg/m^2^) [14].

### Assessment of Exposure to Air Pollution

The PM_2.5_ air pollution dataset originates from the near real-time tracking dataset of atmospheric components in China, known as “*Tracking China Air Pollution*” [15,16,17]. The PM_2.5_ prediction model establishes a 2-tier machine learning framework. In the first tier, the model uses a resampled training data set and the random forest algorithm to predict high-pollution events. In the second-level model, the second random forest model is established using the residuals between the PM_2.5_ concentration simulated by the Community Multiscale Air Quality model and the observed PM_2.5_ concentration. In the 2-level model, a decision tree–based method is used to establish the association between missing data and other parameters, thereby compensating for the absence of satellite data. The PM_2.5_ prediction at a 1 km resolution integrates high-resolution satellite remote sensing aerosol optical depth data and environmental spatial data, such as road networks, to invert the PM_2.5_ concentration at a 1 km resolution, which is fully covered daily. This model effectively captures changes in PM_2.5_ concentration across various spatiotemporal scales and exhibits high accuracy. The cross-validation determination coefficient (CV-R²) ranges from 0.86 to 0.90, indicating strong predictive ability (*R*² ranges between 0.80 and 0.84). To evaluate the model’s ability to detect variations in PM_2.5_ levels in rural areas with a limited number of monitoring stations and on a local scale, national monitoring station data were used for model evaluation. The results indicate that the high-prediction model performs comparably in out-of-bag evaluation, test data evaluation, and yearly cross-validation evaluation, demonstrating extremely high accuracy and robustness. The long-term PM_2.5_ exposure levels are assigned based on the geographical coordinates of the research participants’ permanent residences. The residential address information was collected through questionnaires during the baseline survey. We used the sf package in R software (version 4.4.3; R Foundation for Statistical Computing) to match these addresses with China’s township-level administrative centers’ database of latitude and longitude and assigned the geographical center’s latitude and longitude coordinates of each village to each research participant. All coordinates are part of the WGS84 coordinate system. We then overlaid these coordinates with high-resolution PM_2.5_ raster data, extracted the PM_2.5_ concentration value corresponding to each coordinate point for a specific year and date, and used this as the individual’s long-term exposure level. The total PM_2.5_ concentrations for each participant during 4 different exposure windows were estimated, including moving averages for 1-year, 3-year, 5-year, and 10-year periods before the measurement date.

### Research Outcome

### Statistical Analysis

The composite outcome of this study is the incidence rate of major adverse cardiovascular events. Major adverse cardiovascular events was defined as a composite end point, encompassing the first occurrence of any of the following events: (1) ischemic stroke (IS), (2) coronary heart disease (CHD), (3) acute coronary syndrome (ACS), (4) intracerebral hemorrhage (ICH), and (5) any other form of CVD. In addition to obtaining the relevant disease information for the primary diseases diagnosed by qualified medical institutions (collected through baseline surveys and 10 y follow-up questionnaires), the local CDMS will export data based on ID numbers and match it with our study participants. This CDMS has been developed using the National Basic Public Health Service Management System, which was launched in 2009, and it ensures 100% coverage of all grassroots medical institutions nationwide. If a diagnosis certificate cannot be provided and it is not recorded in the system, it is not considered to have the disease. To assess the impact of PM_2.5_ on specific diseases more specifically, we also included the following outcomes as end points for separate analysis:

- IS: defined as the first hospitalization or emergency event due to ischemic stroke, with the primary diagnosis code being *International Classification of Diseases, Tenth Revision* (*ICD-10*), I63.
- CHD: defined as the first hospitalization or emergency event due to CHD, with the primary diagnosis code being I20-I25. This includes angina pectoris and chronic ischemic heart disease.
- ACS: defined as a severe subtype of CHD, it is characterized by the first hospitalization or emergency event due to ACS, with the primary diagnosis code being I21 (acute myocardial infarction) or I20.0 (unstable angina pectoris).
- ICH: defined as the initial hospitalization or emergency event resulting from ICH. The primary diagnostic code is *ICD-10* I61.
- Any other form of CVD: hospitalization events for other CVD not encompassed by the aforementioned categories (*ICD-10* I00-I99) will be categorized as “other” outcomes and subjected to analysis.

To ensure the accuracy of the outcome definition, we require that each event be coded as the primary diagnosis. For all suspected events, we conduct a secondary confirmation by reviewing the medical records and require that their clinical manifestations and imaging examination results conform to internationally recognized diagnostic standards.

The data were independently entered into the EpiData software (version 3.1; EpiData Consortium) by 2 trained data entry professionals. Any discrepancies between the 2 datasets were resolved through cross-referencing with the original records to ensure data integrity and accuracy. Statistical analyses were performed using R (version 4.4.3; R Foundation for Statistical Computing). Quantitative variables are presented as medians, whereas qualitative variables are summarized as counts (percentages). BMI was calculated as weight (kilogram) divided by height squared (square meter). Fisher exact test and Pearson *χ*^2^ test were used to compare the distribution of categorical variables across groups. The Cox proportional hazards model was used to investigate the association between long-term exposure to PM_2.5_ and the risk of CVD incidence over 1, 3, 5, and 10 years of follow-up, with continuous adjustments for selected covariates. The selection of covariates was based on prior knowledge and known or potential risk factors for CVD. Specifically, these include demographic variables (eg, age, gender, and BMI), socioeconomic factors (eg, educational attainment and household income), behavioral factors (eg, smoking status and alcohol consumption), and clinical history (eg, a history of hypertension). All variables were collected at the baseline of the study and were included as covariates in the multivariate Cox model during the univariate analysis (*P*<.05). The time variable in the model was defined as study time (follow-up time) [18]. For every 1 μg/m^3^ increase in PM_2.5_ concentration, the hazard ratio (HR) and the corresponding 95% CI were estimated. Subgroup analyses were conducted based on demographic and baseline disease risk factors to determine HRs for specific stratifications. Two-sided *P* values less than .05 were deemed statistically significant. On the basis of the tertiles of PM_2.5_ concentration, the population was divided into a low exposure group (Q1), a medium exposure group (Q2 and Q3), and a high exposure group (Q4). The quartile groups were used as ordinal variables, assigned values of 1, 2, 3, and 4, for linear trend tests. The *P* value for trend was calculated to evaluate the overall dose-response trend, and restricted cubic spline analysis was used to examine the curve shape. The interaction *P* value was determined using the likelihood ratio test to assess the modifying effect of PM_2.5_ on disease associations across various subgroups.

## Results

### Characteristics of the Study Participants Included in the Analysis in 2013

The information on the 3 study sites and the study participants included in the analysis is detailed in Table 1. Among the 16,636 eligible participants, 15,745 actually participated in the 10-year follow-up survey, yielding a response rate of 94.64%(15,745/16,636). After excluding 57 participants who lacked geographical location information and 186 participants with incomplete data, 15,502 participants were included in the final analysis (Table 1). The total follow-up period spanned 136,310 person-years. Approximately half of the participants were male (7293/15,502), and 22.76% (3528/15,502) of the participants were aged ≥60 years. The age distribution significantly differed among the 3 study sites (*P*<.001), with a higher proportion of individuals aged ≥60 years in Xiangtan compared to Zhongmu and Longxi (Table 1). Of the participants, 25.96% (4024/15,502) reported current smoking, and 18.35% (2845/15,502) reported alcohol consumption in the past year (Table 1).

**Table 1.** Characteristics of the study population at 2013 baseline survey.

Characteristics^a^	Total	Xiangtan	Longxi	Zhongmu	*P* value for *χ*^2^ test
Total, n (%)	15,502	5279 (34.05)	4971 (32.07)	5252 (33.88)	—^b^
Sex, n (%)					<.001
Female	8209 (52.95)	2770 (52.47)	2863 (57.59)	2576 (49.05)	
Male	7293 (47.05)	2509 (47.53)	2108 (42.41)	2676 (50.95)	
Age (y), n (%)					<.001
<60	11,974 (77.24)	3565 (67.53)	4077 (82.02)	4332 (82.48)	
≥60	3528 (22.76)	1714 (32.47)	894 (17.98)	920 (17.52)	
Education, n (%)					<.001
No schooling	3141 (20.26)	517 (9.79)	1241 (24.96)	1383 (26.33)	
Primary school or higher	12,361 (79.74)	4762 (90.21)	3730 (75.04)	3869 (73.67)	
Marital history, n (%)					<.001
Unmarried	3651 (23.55)	784 (14.85)	1468 (29.53)	1399 (26.64)	
Married	11,098 (71.59)	4173 (79.05)	3298 (66.34)	3627 (69.06)	
Divorced	59 (0.38)	28 (0.53)	17 (0.34)	14 (0.27)	
Widowed	694 (4.48)	294 (5.57)	188 (3.79)	212 (4.03)	
Income (RMB), n (%)^c^					<.001
<6000 (US $887)	11,420 (73.67)	3456 (65.47)	3791 (76.26)	4173 (79.46)	
≥6000 (US $887)	4082 (26.33)	1823 (34.53)	1180 (23.74)	1079 (20.54)	
BMI (kg/m^2^), n (%)					<.001
<18.5	2818 (18.18)	746 (14.13)	1180 (23.74)	892 (16.98)	
≥18.5 to <24	7155 (46.16)	2725 (51.62)	2367 (47.62)	2063 (39.28)	
≥24	5529 (35.66)	1808 (34.25)	1424 (28.64)	2297 (43.74)	
Smoking history, n (%)					<.001
Never	11,478 (74.04)	3574 (67.70)	4008 (80.63)	3896 (74.18)	
Ever (current and former)	4024 (25.96)	1705 (32.30)	963 (19.37)	1356 (25.82)	
Current drinking status, n (%)					<.001
No	12,657 (81.65)	4385 (83.06)	4415 (88.82)	3857 (73.44)	
Yes	2845 (18.35)	894 (16.94)	556 (11.18)	1395 (26.56)	
History of hypertension, n (%)					<.001
No	14,412 (92.97)	4675 (88.56)	3706 (74.55)	5031 (95.79)	
Yes	1090 (7.03)	604 (11.44)	265 (25.45)	221 (4.21)	

a Data might not sum to the total because of missing data.

b Not available.

c Stratification according to total family income/number of people in the household.

### Geographical Distribution of PM_2.5_ Concentration and Regional Differences in the Risk of CVD

**Figure 1.**
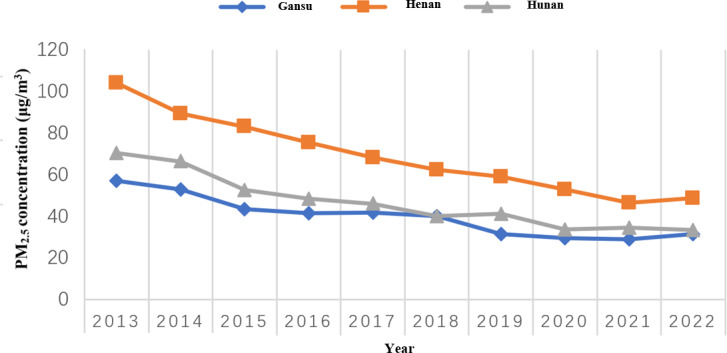
Annual average particulate matter levels at three sites from 2013 to 2022.

The participants were evenly distributed across the 3 sites. From 2013 to 2023, the annual average PM_2.5_ concentrations at these 3 sites exhibited a decreasing trend over time; however, they remained at relatively high levels, ranging from 35.80 to 94.91 μg/m^3^. The median 10-year average PM_2.5_ exposure was 51.50 μg/m^3^. The lowest concentration was found in Gansu Province, whereas the highest was in Henan Province (Figure 1). Over the 10-year period, a total of 628 cases occurred, accounting for 4.05% (628/15,502). Among them, the incidence rates were relatively high among men, individuals aged >60 years, those with an overweight BMI, and those with hypertension (Table 2). For every 1 μg/m^3^ increase in PM_2.5_, the risk of CVD increases by 5% (HR 1.05, 95% CI 1.04‐1.06).

**Table 2.** Analysis of risk factors for cardiovascular and cerebrovascular diseases.

Characteristics	Cases/total, n/N (%)	*P* value for univariate regression	HR^a^^,^^b^ (95% CI)
Total	628/15,502 (4.05)	—^c^	—
PM_2.5_^d^ (μg/m^3^)	—	<.001	1.05 (1.04‐1.06)
Sex		.006	
Female	303/8209 (3.69)		Reference
Male	325/7293 (4.46)		1.25 (0.97‐1.61)
Age (y)		<.001	
<60	304/11,974 (2.54)		Reference
≥60	324/3528 (9.18)		3.57 (2.97‐4.28)
Education		<.001	
Primary school or higher	425/12,361 (3.44)		Reference
No schooling	203/3141 (6.46)		1.19 (0.98‐1.44)
Marital status		.03	
Married	566/11,098 (5.10)		Reference
Unmarried	7/3651 (0.19)		0.07 (0.03‐0.16)
Divorced	1/59 (1.69)		0.41 (0.06‐2.87)
Widowed	54/694 (7.78)		1.20 (0.89‐1.60)
Income^e^ (RMB)		.07	
<6000 (US $887)	473/11,420 (4.14)		1.03 (0.84‐1.26)
≥6000 (US $887)	155/4082 (3.80)		Reference
BMI (kg/m^2^)		<.001	
<18.5	19/2818 (0.67)		0.76 (0.47‐1.23)
≥18.5 to <24	258/7155 (3.61)		Reference
≥24	351/5529 (6.35)		1.35 (1.14‐1.59)
Smoking status		<.001	
Never	382/11,478 (3.33)		Reference
Ever (current and former)	246/4024 (6.11)		1.50 (1.17‐1.90)
Current drinking status		<.001	
No	475/12,657 (3.75)		Reference
Yes	153/2845 (5.38)		1.32 (1.06‐1.64)
History of hypertension		<.001	
No	505/14,412 (3.50)		Reference
Yes	123/1090 (11.28)		2.27 (1.84‐2.80)

a HR: hazard ratio.

b HR analysis included 15,502 participants. HR indicates the increase in incidence risk for every 1 µg/m3 increase in PM_2.5_ concentration.

c Not available.

d PM_2.5_: particulate matter with aerodynamic diameter ≤2.5 μm.

e Stratification according to total family income/number of people in the household.

On the basis of the estimated PM_2.5_ values from satellite remote sensing within a 1 km radius of the research subjects’ residences, the average PM_2.5_ concentration for each year from 2013 to 2022 was calculated, and the changes in PM_2.5_ levels over time for the 3 locations were plotted.

Long-term exposure to varying concentrations of PM_2.5_ over different periods was associated with a slight increase in the incidence of CVD, and this association was statistically significant. Overall, the risk of disease increased significantly with 1-year and 3-year PM_2.5_ exposure, while it slightly decreased with 5-year and 10-year moving average exposure (Table 3). After adjusting for all potential covariates, during the 1-year exposure window, the overall risk of disease increased by 10% for every 1 μg/m^3^ increase in PM_2.5_ (HR 1.10, 95% CI 1.09‐1.11). Specifically, the risk of IS, CHD, ACS, and ICH increased by 13% (HR 1.13, 95% CI 1.11‐1.15), 6% (HR 1.06, 95% CI 1.03‐1.09), 16% (HR 1.16, 95% CI 1.13‐1.19), and 18% (HR 1.18, 95% CI 1.13‐1.24), respectively. These associations were statistically significant (Table 3).

**Table 3.** The association between PM_2.5_^a^ exposure windows and the risk of CVD^b^^,^^c^.

Diseases	1 y		3 y		5 y		10 y	
	HR^d^ (95% CI)	*P* value	HR (95% CI)	*P* value	HR (95% CI)	*P* value	HR (95% CI)	*P* value
CVD	1.10 (1.09‐1.11)	<.001	1.09 (1.08‐1.10)	<.001	1.07 (1.06‐1.08)	<.001	1.05 (1.04‐1.05)	<.001
Ischemic stroke	1.13 (1.11‐1.15)	<.001	1.12 (1.10‐1.14)	<.001	1.10 (1.09‐1.12)	<.001	1.07 (1.06‐1.08)	<.001
Coronary heart disease	1.06 (1.03‐1.09)	.004	1.07 (1.05‐1.09)	.02	1.07 (1.05‐1.09)	.009	1.08 (1.07‐1.09)	<.001
Acute coronary syndrome	1.16 (1.13‐1.19)	<.001	1.15 (1.12‐1.18)	<.001	1.12 (1.10‐1.15)	<.001	1.09 (1.06‐1.11)	<.001
Intracerebral hemorrhage	1.18 (1.13‐1.24)	<.001	1.17 (1.12‐1.22)	<.001	1.14 (1.10‐1.19)	<.001	1.10 (1.07‐1.14)	<.001
Other	1.05 (1.01‐1.09)	.007	1.04 (1.01‐1.08)	.009	1.04 (1.01‐1.07)	.009	1.03 (1.01‐1.05)	.01

a PM_2.5_: particulate matter with aerodynamic diameter ≤2.5 μm.

b CVD: cardiovascular and cerebrovascular diseases.

c Note: Adjusted HRs (95% CIs) of the occurrence risk are presented by per 1 μg/m3 increment in moving average ambient PM_2.5_ concentrations in different durations. The Cox model is adjusted for baseline age, gender, income, education level, BMI, smoking and drinking status, marital status, and history of hypertension.

d HR: hazard ratio.

**Figure 2.**
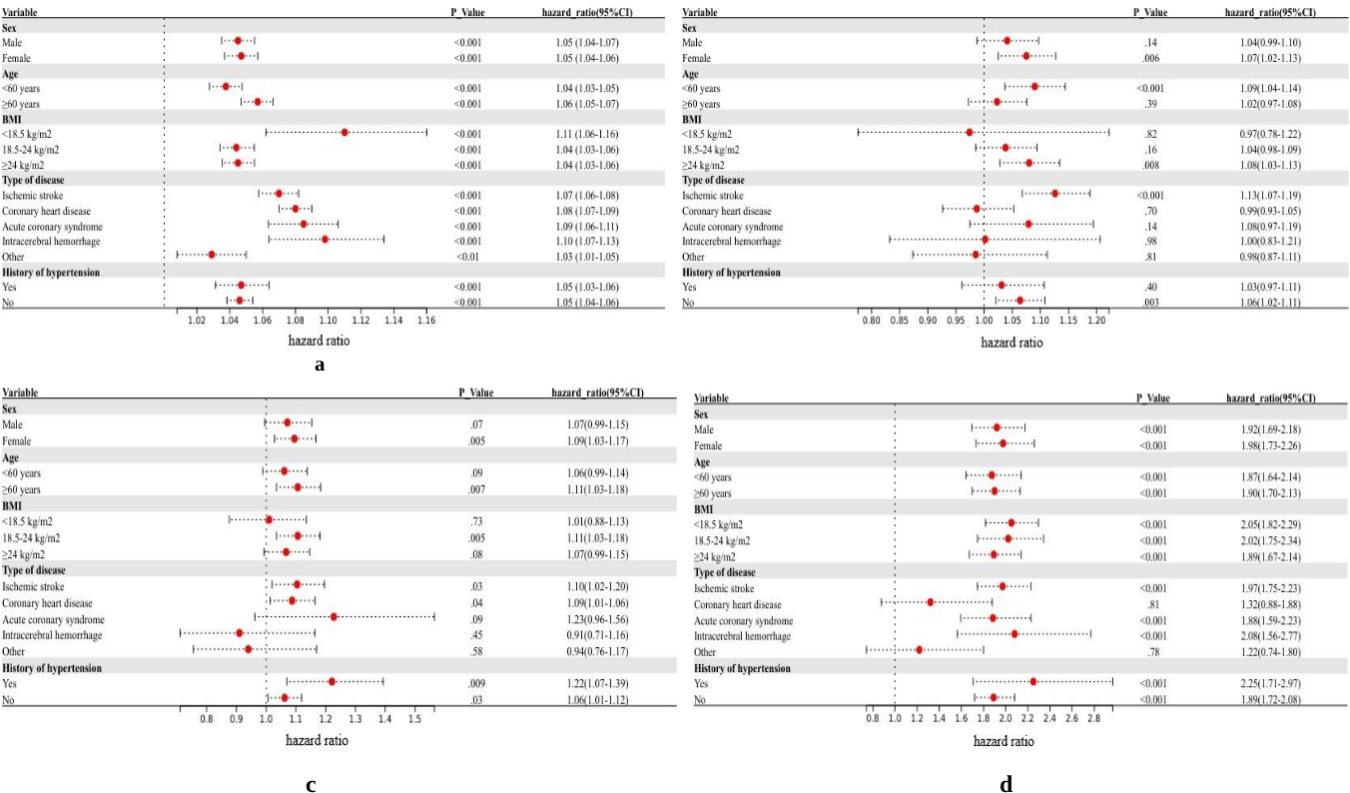
The relationship between particulate matter 2.5 (PM_2.5_) and the risk of cardiovascular and cerebrovascular diseases in different sites. (A) Total, (B) Xiangtan, (C) Longxi, and (D) Zhongmou. The Cox model is adjusted for baseline age, gender, income, education level, BMI, smoking and drinking status, marital status, and history of hypertension.

Stratified analysis by different regions indicated that at the Zhongmu station, on average, the PM_2.5_ concentration was the highest (73.24 μg/m^3^), and its impact on the risk of CVD was higher than that at the Xiangtan and Longxi stations, with an adjusted HR of 1.89 (95% CI 1.73‐2.06; Figure 2). Considering distinct genders, ages, BMI levels, and hypertension histories, the disease risk in the Zhongmu area is notably higher than in other regions. Additionally, at the Xiangtan, Longxi, and Zhongmu research stations, PM_2.5_ exposure was positively correlated with the risk of IS, with adjusted HR of 1.13 (95% CI 1.07‐1.19), 1.10 (95% CI 1.02‐1.20), and 1.97 (95% CI 1.75‐2.23), respectively (Figure 2). Additionally, the risk of ACS in Xiangtan, Longxi, and Zhongmu was 1.08 (95% CI 0.97‐1.19), 1.23 (95% CI 0.96‐1.56), and 1.88 (95% CI 1.59‐2.23), respectively. For ICH, the risks were 1.00 (95% CI 0.83‐1.21), 0.91 (95% CI 0.71‐1.16), and 2.08 (95% CI 1.56‐2.77), respectively.

### Variation of CVD Risk Across PM_2.5_ Concentration Levels

In this study, a fully adjusted model with a 10-year exposure window was used to perform stratified analyses on the association between PM_2.5_ exposure and the risk of CVD across different exposure groups. The research findings indicate that, in comparison to Q1, the risk levels in Q2, Q3, and Q4 showed a gradual increase. The corresponding HRs were 1.05 (95% CI 0.88‐1.26), 1.06 (95% CI 1.01‐1.11), and 1.96 (95% CI 1.67‐2.29), respectively. Furthermore, the trend test yielded statistically significant results (*P* for trend <.001), suggesting the presence of an overall dose-response relationship (Multimedia Appendix 1)*.* The risk was notably higher in Q4 than in the younger population (aged <60 y). It is notable that the exposure concentration among the older population in different regions was higher than that of the younger population (Multimedia Appendix 1), and the interaction effect based on age was statistically significant (interaction *P* value=.03; Table 4).

**Table 4.** Stratified analysis of the association between particulate matter with aerodynamic diameter ≤2.5 μm (PM_2.5_) and the risk of cardiovascular and cerebrovascular diseases.^a^

Characteristics^b^	Quartiles 1 (35.81-44.58μg/m^3^)	Quartiles 2 (44.58-51.50 μg/m^3^)	Quartiles 3 (51.50-68.77 μg/m^3^)	Quartiles 4 (68.77-94.91 μg/m^3^)	Interaction *P* value^c^	*P* value for trend^d^
Total	Reference	1.05 (0.88‐1.26)	1.06 (1.01‐1.11)	1.96 (1.67‐2.29)	—^e^	<.001
Age (y)					.03	
<60	Reference	1.37 (0.97‐1.94)	2.15 (1.49‐3.08)	2.34 (1.70‐3.22)		<.001
≥60	Reference	1.26 (0.94‐1.69)	1.39 (0.91‐2.12)	3.23 (2.42‐4.30)		<.001
Sex					.96	
Male	Reference	1.28 (0.93‐1.76)	1.74 (1.20‐2.52)	2.63 (1.94‐3.55)		<.001
Female	Reference	1.38 (1.01‐1.88)	1.81 (1.23‐2.68)	2.65 (1.95‐3.60)		<.001
BMI (kg/m^2^)					.02	
<18.5	Reference	0.85 (0.67‐1.03)	—^f^	3.07 (2.04‐4.12)		<.001
≥18.5 to <24	Reference	1.26 (0.92‐1.72)	1.31 (0.81‐2.11)	2.62 (1.91‐3.61)		<.001
≥24	Reference	1.41 (1.01‐1.96)	2.26 (1.60‐3.21)	2.76 (2.04‐3.75)		<.001
Education					<.001	
Primary school or higher	Reference	1.23 (0.96‐1.58)	1.90 (1.39‐2.59)	1.82 (1.39‐2.38)		<.001
No schooling	Reference	1.65 (1.00‐2.71)	1.99 (1.16‐3.43)	5.65 (3.75‐8.50)		<.001
Income^g^ (RMB)					.10	
<6000 (US $887)	Reference	1.33 (1.01‐1.74)	1.70 (1.24‐2.33)	2.89 (2.26‐3.69)		<.001
≥6000 (US $887)	Reference	1.31 (0.87‐1.95)	2.18 (1.30‐3.66)	1.93 (1.22‐3.05)		<.01
Smoking status					.85	
Never	Reference	1.38 (1.04‐1.84)	1.89 (1.34‐2.66)	2.63 (2.00‐3.46)		<.001
Ever (current and former)	Reference	1.25 (0.87‐1.79)	1.69 (1.10‐2.61)	2.73 (1.94‐3.85)		<.001
Current drinking status					.11	
No	Reference	1.29 (1.01‐1.65)	1.46 (1.05‐2.02)	2.64 (2.09‐3.35)		<.001
Yes	Reference	1.64 (0.92‐2.92)	3.01 (1.72‐5.28)	3.10 (1.85‐5.20)		<.001
History of hypertension					.66	
Yes	Reference	1.21 (1.05‐1.39)	0.90 (0.77‐1.05)	1.60 (1.35‐1.89)		<.001
No	Reference	1.32 (1.02‐1.71)	1.89 (1.41‐2.53)	2.65 (2.09‐3.37)		<.001

a The Cox model is adjusted for baseline age, gender, income, education level, BMI, smoking and drinking status, marital status, and history of hypertension.

b Data might not sum to the total because of missing data.

c Interaction *P* value from likelihood ratio tests.

d *P* for trend calculated by treating PM_2.5_ as an ordinal variable (Q1-Q4).

e Not applicable.

f There were no results in the subgroup analysis, which was due to the model not converging because of the small number of patients.

g Stratification according to total family income/number of people in the household.

## Discussion

### Principal Findings

The results of this multicenter, population-based, prospective study conducted in rural areas of China indicate a significant association between long-term exposure to PM_2.5_ and an increased risk of CVD. In particular, the correlation between long-term exposure to PM_2.5_ (over periods of 1 and 3 y) and disease risk was more pronounced, even after accounting for known risk factors. Notably, there were regional variations and dose-response trends in the risk of disease occurrence at different levels of PM_2.5_ exposure.

The epidemiological evidence of the association between PM_2.5_ and CVD revealed in this study significantly corroborated the findings of multicenter cohort studies conducted worldwide [19]. At the same time, it also highlighted the particularity of rural exposure. A cross-national prospective cohort study [20] has revealed that long-term exposure to outdoor PM_2.5_ is associated with an increased risk of CVD in adults aged between 35 and 70 years. For every 10 µg/m^3^ increase in PM_2.5_ exposure, the risk of CVD death, CVD events, myocardial infarction, and stroke increased by 3%, 5%, 3%, and 8%, respectively [21]. Furthermore, the study revealed that the risk of developing CVD was higher in rural areas compared to urban areas. In our current research, similar results were also observed. In particular, the correlation between 1 to 3 years of PM_2.5_ exposure and disease risk was more pronounced, with regional variations. This may be related to the following reasons: First, short-term exposure could rapidly trigger CVD events through acute inflammation and oxidative stress mechanisms, whereas long-term exposure could exacerbate the occurrence risk of atherosclerosis and CVD through cumulative effects [20]. A meta-analysis conducted by Jeroen de Bont et al [1] revealed that short-term exposure to PM_2.5_ is associated with an increased risk of hypertension, stroke, and myocardial infarction. Long-term exposure to PM_2.5_ was largely related to an increased risk of atherosclerosis, myocardial infarction, hypertension, stroke, and stroke mortality [22]. Second, during the study period, some participants may have relocated to new residences, which could lead to misclassification bias in the exposure assessment, potentially reducing the risk ratio. Finally, during the follow-up period, study participants may have died from causes other than CVD, particularly in long-term follow-ups, which could weaken the estimation of CVD risk. Furthermore, our study demonstrated that the effects of PM_2.5_ on specific CVD differed significantly both in the overall population and in regional stratified analyses. This regional heterogeneity may arise from the complex interplay of multiple mechanisms. The variations in PM_2.5_ concentrations and chemical compositions across different regions contribute to the observed disparities. The most polluted areas, such as Henan, were characterized by high population densities and an energy structure that heavily relied on coal combustion, leading to persistent high levels of air pollution from coal burning [23]. The resulting sulfate and nitrate particles made up over 40% of the PM_2.5_ mass concentration. Additionally, the high-salt dietary habits prevalent in Henan, combined with prolonged exposure to PM_2.5_, may have increased the risk of hypertension and vascular damage [24]. Collectively, these factors contributed to an increased incidence of CVD. In our study, the results of trend tests and restricted cubic spline analysis indicated a dose-response relationship between PM_2.5_ exposure and overall CVD risk. However, this relationship was not strictly linear. These findings suggest that the impact of PM_2.5_ on CVD may exhibit a “*threshold effec*t” [25], whereby its toxic effects become markedly pronounced upon exceeding a certain threshold [26]. Evidence suggests that at lower concentrations, cellular autophagy can remove damaged organelles and maintain cellular homeostasis. This compensatory mechanism may partially mitigate the toxic effects of PM_2.5_ on cells, thereby reducing the associated risk [22]. However, when PM_2.5_ concentrations were excessively high, the oxidative stress induced by PM_2.5_ increased in a dose-dependent manner, leading to a significant rise in ROS and cytochrome C expression in vascular endothelial cells. This cascade activates caspase-3, ultimately resulting in DNA fragmentation and cell apoptosis [27]. Furthermore, exposure to high concentrations of PM_2.5_ can trigger multiple programmed cell death pathways in vascular endothelial cells, disrupt tight junction proteins, impair endothelial cell integrity, and consequently cause further damage to cardiovascular tissues, significantly elevating the risk of CVD [28].

Various biological mechanisms have been proposed to explain the association between PM_2.5_ exposure and CVD events, including increased systemic inflammation and oxidative stress, accelerated atherosclerosis, and changes in cardiac autonomic nerve function [29-32]. Exposure to particulate matter is associated with an increased risk of heart disease, primarily through the initiation and promotion of atherosclerosis progression, which underlies the majority of CVD [33]. Exposure to PM_2.5_ has been shown to increase the levels of ROS. The subsequent accumulation of ROS exacerbates oxidative stress, leading to cellular and molecular damage, including DNA, proteins, and lipids [34]. Additionally, exposure to PM_2.5_ promoted the secretion of inflammatory cytokines, leading to endothelial cell activation and a series of pathological changes in the vascular endothelium, thereby fostering the development of CVD [35].

The older population is considered a vulnerable group, susceptible to a range of factors, including immune aging, comorbidities, and environmental influences. Consequently, research focused on this high-risk demographic is of paramount importance. The risk of CVD was elevated in the high-exposure group, whereas it was marginally reduced in the medium and low exposure groups relative to younger individuals. At moderate to low exposure levels, the older population’s cumulative physiological compensatory capacity can partially counteract the damage caused by pollution, temporarily maintaining the homeostasis of their internal environment [32]. As previously mentioned, prolonged exposure to high levels results in a substantial rise in the production of reactive oxygen species [34]. This increase surpasses the antioxidant capacity of older people, which is already compromised, thereby exacerbating oxidative damage [36]. Although most studies and expert consensus have reached the conclusion that PM_2.5_ can increase the risk of CVD, it is worth noting that some studies have failed to find a relationship between PM_2.5_ and the risk of CVD [37,38]. These findings underscore the intricate and uncertain dynamic relationship between air pollution and the health outcomes of the older population, emphasizing the need for further research to accurately assess the impact of PM_2.5_ on disease incidence risk and mortality within this demographic.

### Limitations

First, the estimated exposure concentrations for each participant were derived from the baseline survey conducted in 2013. During the follow-up period, participants who moved away were excluded from the analysis, as we lacked comprehensive migration histories for these individuals. This may result in selection bias. Migration patterns in rural areas were typically linked to younger age, higher socioeconomic status, and superior health conditions. This could diminish the gradient between exposure levels and disease risk, potentially leading to an underestimation of the true health impacts of PM_2.5_. Future cohort studies should implement more comprehensive tracking systems to gather exposure data on migrants, thereby minimizing such selection bias as much as possible. Second, we did not evaluate the indoor PM_2.5_ concentration. Ideally, individual exposure should encompass both indoor and outdoor components. Due to the absence of indoor exposure data, we used the outdoor concentration as a proxy variable, which may result in exposure misclassification. In rural areas of China, the use of solid fuels, such as coal and biomass, may exhibit a spatial correlation with outdoor PM_2.5_ concentrations. On the other hand, factors such as rural ventilation practices and house structure can influence the relationship between indoor and outdoor concentrations, thereby increasing the complexity of exposure assessment. Considering these factors, unmeasured indoor exposure was more likely to diminish rather than amplify the risk ratio we reported. Future research can supplement the collection of indoor PM_2.5_ data and develop individual exposure models that integrate indoor and outdoor monitoring to more accurately analyze the impact of indoor pollution sources on individual exposure. Third, we did not adjust for any other key air pollutants in the model. Both NO_2_ and SO_2_ were strong respiratory irritants that severely damage the respiratory and cardiovascular systems [39]. The interaction with PM_2.5_ may result in a synergistic effect. Owing to the lack of sufficient precise data on air pollution exposure at that time, it may have affected our interpretation of the specific effects of PM_2.5_. However, there was a moderate to high spatial correlation among air pollutants, and including all of them in a multivariate model may lead to collinearity issues [40]. In the analysis of multipollutant models, the effect of PM_2.5_ was typically the most robust [31]. Simultaneously, PM_2.5_ possesses direct cardiovascular toxicity, and its role as a key risk factor is independent. Future research could collect more comprehensive pollutant data at individual exposure levels to more accurately assess the health effects of PM_2.5_. Finally, although we adjusted for several potential confounding factors in the multivariate model, it was impossible to completely eliminate confounding bias due to the presence of unknown and unmeasured residual confounding factors.

Despite these limitations, our research was enhanced by using data from a large-scale, population-based prospective cohort study that featured a 10-year follow-up period and high-quality outcome assessments. Short-term fluctuations in PM_2.5_ can have detrimental health effects, yet long-term exposure may have more significant clinical health implications on CVD morbidity and mortality, as individuals are usually exposed to higher levels of air pollution over an extended period [31]. One significant advantage of this study is that it focuses on rural areas in China, covering a wide range of PM_2.5_ concentrations (ranging from 35.81 to 94.91 µg/m^3^), and conducts risk assessments for different exposure windows of specific CVD, thereby laying a solid scientific foundation for evaluating the exposure-response relationship. The generalizability of the results of this study to other rural populations requires careful consideration. The prospective design, large sample size, and detailed assessment of exposure and confounding factors have enhanced the reliability of the conclusions. Therefore, it is likely that they are applicable to rural populations facing similar environments. However, when extending the results to rural populations with significantly different pollution sources and lifestyles, caution is necessary. Future research to validate our model in other rural environments will help confirm the external validity of these associations.

### Conclusions

Our research findings indicate that long-term exposure to PM_2.5_ was significantly associated with an elevated risk of CVD among rural populations, and this association exhibited regional variations. In regions with high levels of PM_2.5_ pollution, comprehensive measures and strategies aimed at reducing air pollution and enhancing public awareness of self-protection should be implemented to mitigate the associated disease risks. This discovery could enhance our understanding of the potential public health risks associated with PM_2.5_ exposure and underscore the important role of environmental governance in promoting public health outcomes. Furthermore, future research efforts should concentrate on clarifying the impacts of PM_2.5_ exposure on the health of various population groups and the underlying mechanisms, thereby contributing to the development of comprehensive intervention measures to mitigate the negative effects of air pollution on public health.

## Supplementary material

10.2196/81218 Multimedia Appendix 1 The nonlinear relationship between particulate matter with aerodynamic diameter ≤2.5 μm and cardiovascular and cerebrovascular diseases and the exposure levels of the older adult population.

10.2196/81218 Checklist 1 STROBE cohort checklist.

## Abbreviations

ACS: acute coronary syndrome
CDMS: chronic disease management system
CHD: coronary heart disease
CVD: cardiovascular and cerebrovascular diseases
HR: hazard ratio
*ICD-10*: *International Classification of Diseases, Tenth Revision*
ICH: intracerebral hemorrhage
IS: ischemic stroke
PM_2.5_: particulate matter 2.5
