# Supplementary material for: Long-Term Ambient Air Pollution Exposure and the Risk of Cardiovascular and Cerebrovascular Diseases in Rural Chinese Populations: 10-Year Follow-Up of a Multicenter Prospective Cohort Study
Source: JMIR Public Health Surveill. 2025 Nov 28;11:e81218. doi: 10.2196/81218 (PMC12669913; doi:10.2196/81218)
Supplement: Multimedia Appendix 1 [file publichealth-v11-e81218-s001.docx]

*Supplementary Materials：*

*eTable 1：Average levels of PM_2.5_ exposure for different age groups*

| *Site* | *Age* | | *P value ^a^* |
| --- | --- | --- | --- |
|  | *<60 years* | *≥60 years* |  |
| *Xiangtan* | 49.91 **μg**/m^3^ | 51.82 **μg**/m^3^ | <.001 |
| *Longxi* | 43.82 **μg**/m^3^ | 44.81 **μg**/m^3^ | <.001 |
| *Zhongmu* | 72.63 **μg**/m^3^ | 76.13 **μg**/m^3^ | <.001 |

^a^ The *P-*value is a statistical indicator used to determine whether there is a significant difference in PM_2.5_ levels among different age groups through variance analysis.


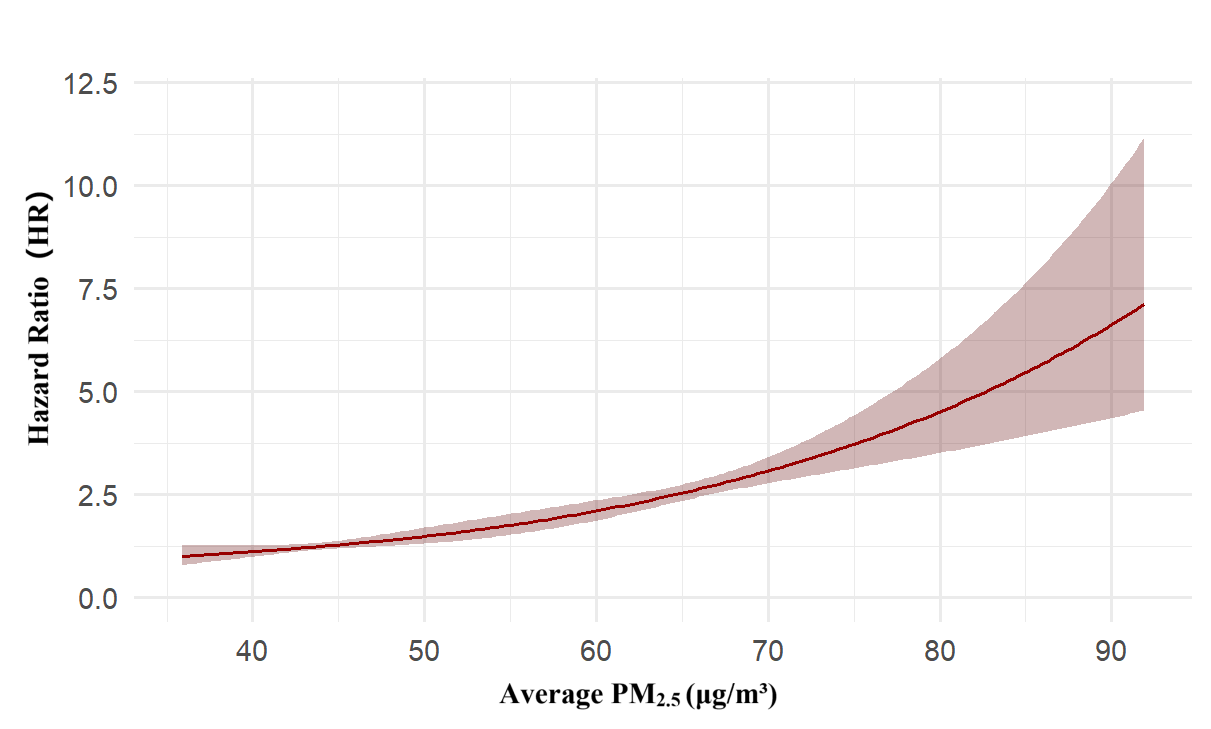


*eFigure 1. The increased risk of CVD along with the increasing average level of PM_2.5_*

The restricted cubic spline curve was adjusted by the Cox model for baseline age, gender, income, education level, BMI, smoking and drinking status, marital status, and history of hypertension. The red curve and the shaded area represent the predicted HR and 95% CI respectively. The reference PM_2.5_ concentration was 35.81(μg/m³), and non-linear *p-*value was significant (*P* < .001).

*eTable 2：Likelihood ratio test results of the restricted cubic spline model*

| *Number of knots* | *Likelihood ratio test chi-square value (χ^2^)* | *P value* |
| --- | --- | --- |
| *3 knots* | 119.76 | <.001 |
| *4 knots* | 341.54 | <.001 |

*eTable 3：List of abbreviation*

| *Abbreviation* | *Definition* |
| --- | --- |
| *CVD* | *Cardiovascular and Cerebrovascular Diseases* |
| *Particulate Matter* | PM_2.5_ |
| *GBD* | Global Burden of Disease |
| ESCAPE | European Study of Cohorts for Air Pollution Effects |
| LMICs | Low- and Middle-Income Countries |
| WHO-AQG | WHO Air Quality Guidelines |
| CDMS | **Chronic Disease Management System** |
| *BMI* | Body Mass Index |
| *TAP* | Tracking China Air Pollution |
| *CMAQ* | Community Multiscale Air Quality |
| *AOD* | Aerosol Optical Depth |
| *MACE* | **Major Adverse Cardiovascular Events** |
| *IS* | **Ischemic Stroke** |
| *CHD* | **Coronary Heart Disease** |
| *ACS* | **Acute Coronary Syndrome** |
| *ICH* | **Intracerebral Hemorrhage** |
| *CDMS* | **Chronic Disease Management System** |
| *HR* | Hazard Ratio |
| *CI* | Confidence Interval |
| *RCS* | Restricted Cubic Spline |
| *ROS* | Reactive Oxygen Species |

*eTable 4：Comparison of the characteristics of the study population among different groups*

| *Characteristics^a^* | *Case group (n=628)* | *Non-case group (14874)* | *P for χ^2^ test* |
| --- | --- | --- | --- |
| *Total, n(%)* | 5279 (34.1%) | 4971 (32.1%) | NA |
| *Sex, n(%)* |  |  | .018 |
| Female | 303 (48.2%) | 7906 (53.2%) |  |
| Male | 325 (51.8%) | 6968 (49.8%) |  |
| *Age, n(%)* |  |  | <.001 |
| <60 years | 304 (48.4%) | 11670 (78.5%) |  |
| ≥60 years | 324 (51.6%) | 3204 (21.5%) |  |
| *Education, n(%)* |  |  | <.001 |
| No schooling | 203 (32.3%) | 2938 (19.8%) |  |
| Primary school or higher | 425 (67.7%) | 11936 (80.2%) |  |
| *Marital history, n(%)* |  |  | <.001 |
| Unmarried | 2 (0.3%) | 75 (0.5%) |  |
| Married | 582 (92.7%) | 14265 (95.9%) |  |
| Divorced | 0 (0.0%) | 14 (0.1%) |  |
| Widowed | 44 (7.0%) | 520 (3.5%) |  |
| *Income, n(%)^b^* |  |  | .362 |
| <6000 RMB | 473 (75.3%) | 10947 (73.6%) |  |
| ≥6000 RMB | 155 (24.7%) | 3927 (26.4%) |  |
| *BMI, n(%)* |  |  | <.001 |
| <18.5 kg/m^2^ | 19 (3.0%) | 2799 (18.8%) |  |
| ≥18.5 to <24 kg/m^2^ | 258 (41.1%) | 6897 (46.4%) |  |
| ≥24 kg/m^2^ | 351 (55.9%) | 5178 (34.8%) |  |
| *Smoking history, n(%)* |  |  | <.001 |
| Never | 382 (60.8%) | 11096 (74.6%) |  |
| Ever(current and former) | 246 (39.2%) | 3778 (25.4%) |  |
| *Current drinking status, n(%)* |  |  | <.001 |
| No | 475 (75.6%) | 12182 (81.9%) |  |
| Yes | 153 (24.4%) | 2692 (18.1%) |  |
| *History of hypertension, n(%)* |  |  | <.001 |
| No | 505 (80.4%) | 13907 (93.5%) |  |
| Yes | 123 (19.6%) | 967 (6.5%) |  |

Abbreviations: NA=not available; BMI=body-mass index; RMB=RenMinBi.

^a^ Data might not sum to total because of missing data.

^b^Stratification according to total family income/number of people in the household.
